# Influence of PEDOT:PSS crystallinity and composition on electrochemical transistor performance and long-term stability

**DOI:** 10.1038/s41467-018-06084-6

**Published:** 2018-09-21

**Authors:** Seong-Min Kim, Chang-Hyun Kim, Youngseok Kim, Nara Kim, Won-June Lee, Eun-Hak Lee, Dokyun Kim, Sungjun Park, Kwanghee Lee, Jonathan Rivnay, Myung-Han Yoon

**Affiliations:** 1 School of Materials Science and Engineering, Gwangju Institute of Science and Technology, Gwangju, 61005 Republic of Korea; 2 Research Institute for Solar and Sustainable Energies, Gwangju Institute of Science and Technology, Gwangju, 61005 Republic of Korea; 3 RIKEN Center for Emergent Matter Science (CEMS), 2-1 Hirosawa, Wako, Saitama 351-0198 Japan; 4 Heeger Center for Advanced Materials, Gwangju Institute of Science and Technology, Gwangju, 61005 Republic of Korea; 5 Department of Biomedical Engineering, Northwestern University, Evanston, IL 60208 USA; 6 Present Address: Laboratory of Organic Electronics, ITN, Linköping University, Norrköping, SE-601 74 Sweden; 7 Present Address: Department of Electronic Engineering, Gachon University, Seongnam, 13120 Republic of Korea

## Abstract

Owing to the mixed electron/hole and ion transport in the aqueous environment, poly(3,4-ethylenedioxythiophene):poly(styrenesulfonate)-based organic electrochemical transistor has been regarded as one of the most promising device platforms for bioelectronics. Nonetheless, there exist very few in-depth studies on how intrinsic channel material properties affect their performance and long-term stability in aqueous environments. Herein, we investigated the correlation among film microstructural crystallinity/composition, device performance, and aqueous stability in poly(3,4-ethylenedioxythiophene):poly(styrenesulfonate) films. The highly organized anisotropic ordering in crystallized conducting polymer films led to remarkable device characteristics such as large transconductance (∼20 mS), extraordinary volumetric capacitance (113 F·cm^−3^), and unprecedentedly high [μC^*^] value (∼490 F·cm^−1^V^−1^s^−1^). Simultaneously, minimized poly(styrenesulfonate) residues in the crystallized film substantially afforded marginal film swelling and robust operational stability even after >20-day water immersion, >2000-time repeated on-off switching, or high-temperature/pressure sterilization. We expect that the present study will contribute to the development of long-term stable implantable bioelectronics for neural recording/stimulation.

## Abstract (web summary)

The lack of understanding of mixed transport in ion-permeable conjugated polymer films hinders the advance of organic electrochemical transistors for bioelectronics. Here, the authors elucidate the structure-property-performance relationships for conventional and crystallized PEDOT:PSS films.

## Introduction

While the past decade has witnessed remarkable advances in the field of organic bioelectronics^1–6^, organic electrochemical transistors (OECTs) have been regarded as one of the most promising device platforms for this purpose^7^. An OECT is a type of transistor where the source-to-drain current is electrochemically modulated by applying biases on the gate electrode^8,9^. In comparison with other organic electronic devices, OECTs have several advantages, including simple device fabrication, strechability, relatively low operation voltages, and decent on-off current ratios^10^. Accordingly, many researchers have developed various types of OECT-based bioelectronics with the capability of sensing DNAs^11^, hormones^12^, metabolites^13^, and neurotransmitters^14^, or of monitoring cells^15,16^, tissues^17^, or brain activities^18^.

To understand the mechanism of OECT device operation, the mixed transport of holes/electrons and ions through an organic channel should be considered simultaneously^19^. When an electrical bias is applied to the gate electrode, the conductivity of the organic layer is controlled by driving small cations (or anions) from the electrolyte medium to the channel layer, thereby dedoping (or doping) the constituent organic conductor, resulting in the efficient modulation of source-to-drain current^20^. In this regard, OECTs employ the whole volume of organic film as an effective channel, unlike typical organic field-effect transistors (OFETs) where the interface between semiconducting and dielectric layers functions as a major channel. From the perspective of engineering the channel microstructure, in-plane π–π stacking among the conjugated moieties, as well as well-organized out-of-plane ordering, is highly desired to facilitate both intra- and interchain transport of charge carriers along the channel direction^21,22^. Meanwhile, porosity with micro/nanoscopic voids and molecular-scale dispersion of ion-conductive moieties (e.g., polyelectrolytes or ion-conductive side chains) should be uniformly distributed throughout the organic layer to enable facile permeation of small ions into the channel layer (e.g., conjugated molecules or polymers), leading to an effective control over charge carrier density^19,23,24^.

Among a variety of soft electronic materials, poly(3,4-ethylenedioxythiophene) doped with poly(styrenesulfonate) (PEDOT:PSS) has been one of the most frequently used channel materials for OECTs and related bioelectronic devices, owing to its high electrical conductivity, good optical transparency, and decent biocompatibility^13,17,18,25^. In addition, conductive films as well as complicated devices based on PEDOT:PSS can be easily fabricated via a series of solution-based processes using commercially available aqueous PEDOT:PSS solution, wherein hydrophobic short PEDOT chains are aggregated in the form of nanoparticles wrapped with hydrophilic long PSS chains, and a certain portion of deprotonated styrene sulfonate units are in close proximity to conjugated EDOT moieties as dopants^26^. In principle, the long PSS chains may occupy a substantial volume of the channel layer and/or disturb the PEDOT chain arrangement suitable for efficient charge transport, resulting in low film conductivity and poor OECT performance far from the optimal metrics achievable with PEDOT:PSS in theory. Furthermore, the residual amount and relative allocation of hydrophilic PSS in the bulk PEDOT:PSS film need to be judiciously controlled, since it is well known that polyelectrolytes such as PSS are prone to unintentional swelling in the presence of water^27,28^. For this reason, chemical cross-linkers (e.g., 3-glycidoxypropyltrimethoxysilane: GOPS) have been commonly employed to improve aqueous stability of PEDOT:PSS devices and the GOPS-blended PEDOT:PSS has been widely used for OECT devices^18,29,30^. However, chemical cross-linking densifies PEDOT:PSS films and interferes with interchain charge transport, leading to the significant decrease in both electronic and ionic mobilities^31–33^. Therefore, we can infer that stable device performance, and thus prolonged operational reliability of OECTs, can be substantially improved by strictly controlling the microstructural crystallinity of hydrophobic conjugated moieties (i.e., PEDOT) and the amount/allocation of hydrophilic moieties (i.e., PSS) in the channel layer, particularly for aqueous electrolyte conditions and bioelectronic applications. Several research groups have reported on practical issues in organic bioelectronics including aqueous stability^24,34–37^, but there exist few in-depth studies addressing the aqueous stability of the OECT channel material itself (without chemical cross-linking) and device performance under critical stress conditions, nor have any studies looked at correlating the observed phenomena with the microstructural crystallinity and composition of constituent organic conductors.

Herein, we report the close interdependence of film microstructural crystallnity/composition, OECT device performance, and aqueous stability, using pristine, ethylene glycol-treated (EG-P), and crystallized PEDOT:PSS (Crys-P) films, all of which are composed of only PEDOT and PSS without chemical crosslikers such as GOPS. First, the detailed microstructures and the relative PSS compositions in three types of PEDOT:PSS films were carefully examined via grazing-incidence wide-angle X-ray scattering (GIWAXS) and X-ray photoelectron spectroscopy (XPS) in a comparative manner. Then, OECT devices based on EG-P and Crys-P were prepared by conventional lithography and characterized electrically so that the corresponding device performance parameters such as transconductance and contact resistance could be measured, in order to understand the correlation between the film microstructural crystallnity/composition and (horizontal) charge transport along the channel direction. Moreover, electrochemical impedance spectroscopy was performed to estimate the volumetric capacitance of the Crys-P and EG-P layers and address the structural/compositional effect on (vertical) ion penetration and thus PEDOT dedoping. Finally, the aqueous stability of Crys-P/EG-P films and OECT device operation were examined under several harsh stress conditions and correlated with the corresponding film microstructure/composition.

## Results

### Microstructures and compositions in PEDOT:PSS films

**Fig. 1:**
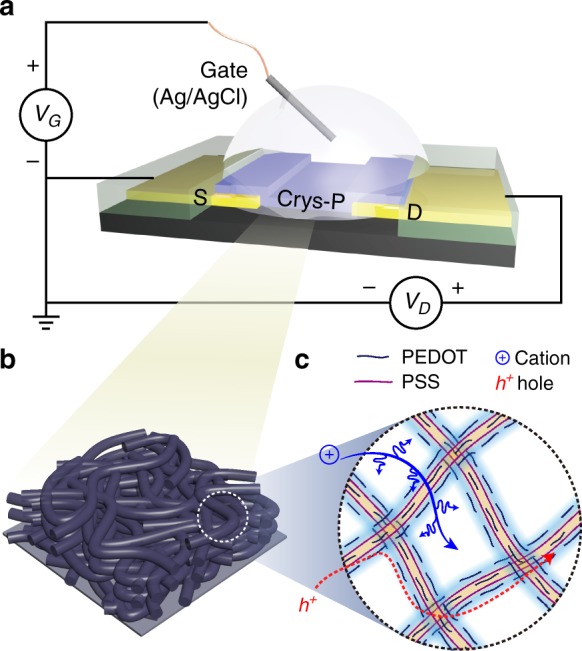
Crystallized PEDOT:PSS (Crys-P) OECTs. **a** An illustration of the device configuration of Crys-P OECT. **b** A schematic diagram of the Crys-P film microstructure and **c** the hole and ion transport therein

**Fig. 2:**
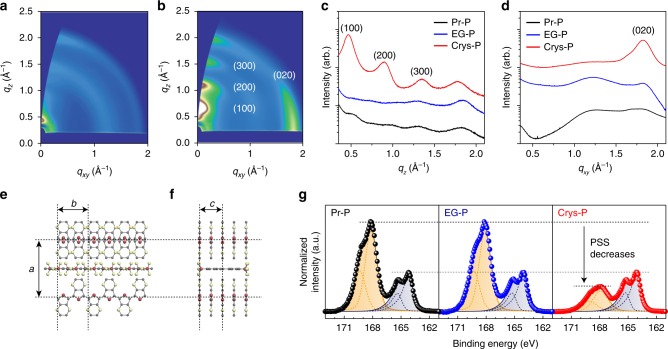
Film microstructures and compositions in PEDOT:PSS films. **a** Two-dimensional grazing incidence wide-angle x-ray scattering (GIWAXS) patterns obtained for 5% ethylene glycol-treated PEDOT:PSS (EG-P) and **b** crystallized PEDOT:PSS (Crys-P) films. **c** Vertical and **d** horizontal GIWAXS profiles of pristine PEDOT:PSS (Pr-P), EG-P, and Crys-P films, where *q*_*z*_ and *q*_*xy*_ are the perpendicular and parallel wave vector transfers with respect to sample surface, respectively. **e**, **f** A schematic illustration of the proposed polymer chain arrangement in the Crys-P film in (**e**) the c-axis and (**f**) the b-axis directions. The a-axis direction is perpendicular to the substrate surface (a-axis direction). **g** X-ray S *2p* photoelectron spectra of Pr-P, EG-P, and Crys-P films. The deconvoluted profiles were fitted with two symmetric/asymmetric Gaussian-Lorenzian functions representing sulfur atoms from styrene sulfonate (SS, yellow gradients) and EDOT thiophene (EDOT, navy gradients)

In this research, crystallized PEDOT:PSS (Crys-P) film was prepared by solvent-assisted crystallization^38^ and utilized as an OECT channel layer after patterning (Fig. 1). First, the microstructure of the Crys-P film was examined with GIWAXS and compared with that of pristine PEDOT:PSS (Pr-P) and ethylene glycol-treated PEDOT:PSS (EG-P) films^39^. Pr-P and EG-P films were chosen as control for the comparison with the more structurally- and compositionally-controlled PEDOT:PSS (i.e., Crys-P). Particularly, the ethylene glycol-treatment is a commonly used method for fabricating PEDOT:PSS OECTs with the improved device characteristics^39^ and EG-P was prepared without chemical cross-linking in this manner. Note that the EG-P exhibits higher electrical conductivity than Pr-P via the phase segregation of surplus PSS and the improvement of film crystallinity^40,41^. Indeed, as shown in Fig. 2 and Supplementary Figure 1, EG-P showed more prominent peaks at *q* = 1.2 Å^−1^ (known as PSS halo) and ~1.8 Å^−1^ (π–π stacking in PEDOT) than Pr-P, which is in accordance with the results in the previous literature^19,42^. More noticeably, the Crys-P film exhibited much enhanced crystallinity and highly anisotropic molecular ordering compared to Pr-P and EG-P films (Fig. 2a, b, see also Supplementary Fig. 1). In the case of Crys-P, the vertical GIWAXS profile shows very clear Bragg progressions (*h*00) due to lamellar stacking of PEDOT:PSS along the out-of-plane direction (*q*_*z* = _0.45, 0.90, and 1.35 Å^−1^ for *d* = 1.40_(100)_, 0.70_(200)_, and 0.35_(300)_ nm, respectively; Fig. 2c)^43,44^. Referring to the out-of-plane *d*-spacing of the single molecule-doped PEDOT (~ 1.4 nm)^45^, we infer that flattened PSS chains are alternately stacked with PEDOT stacking layers (Fig. 2e and f). Furthermore, its horizontal GIWAXS profile contains a strong peak (*q*_*xy*_ = 1.8 Å^−1^ for *d* = 0.34_(020)_ nm; Fig. 2d), which can be assigned to the π–π stacking of PEDOT along the in-plane direction (Fig. 2d)^26,45^. In contrast, the isotropic broad humps appearing at *q* = 1.2 Å^−1^ in Pr- and EG-P films can be attributed to the separated domain of randomly distributed PSS^26,40,46^. The structural investigation results indicate that the Crys-P film features significantly enhanced lamellar stacking, which is rearranged perpendicular to the substrate, and increased crystalline domain size, in particular the preferential edge-on stacking of PEDOT chains (Fig. 2e, f). Such an edge-on polymer alignment could be beneficial for enhancing the performance of an OECT device by facilitating the interchain transport of charge carriers in the in-plane direction^22,47^ (vide infra). It is also noteworthy that the aforementioned anisotropic alignment of conjugated polymer backbones in Crys-P is reminiscent of the highly organized microstructure of a vapor-phase polymerized PEDOT film doped with p-toluenesulfonate (PEDOT:Tos), suggesting that solution-based polymer film deposition followed by solvent-assisted crystallization can create a similar film microstructure^40,45^.

Next, the relative amount of PSS to PEDOT was estimated using XPS to investigate the variation of chemical composition in Pr-P, EG-P, and Crys-P films. As shown in Fig. 2g, raw XPS data sets were acquired using three types of PEDOT:PSS films in the range of 161–173 eV (S 2*p*), and fitted with two symmetric/asymmetric Gaussian-Lorentzian functions, representing sulfur atoms from (i) the styrene sulfonate (SS, 171–167 eV; yellow gradients) and (ii) the EDOT thiophene (EDOT, 167–163 eV; navy gradients)^48,49^. Considering the molecular weight of each unit, the fitting results normalized with the number of EDOT sulfur atoms, indicate that the Crys-P film contains a significantly lower molar ratio of SS to EDOT units (*M*_SS_: *M*_EDOT_ = 0.6) than the Pr-P film (1.8). On the other hand, the EG-P film showed almost the same composition as the Pr-P film. This reduced metric of SS in Crys-P was also confirmed by thermogravimetric analysis (TGA). As shown in Supplementary Figure 2, Crys-P exhibited a significantly decreased weight loss between 30 and 100 °C (8.5% of initial weight), which can be attributed to the reduced evaporation of water molecules held by hydrophilic sulfonate groups^27^, in comparison with Pr-P (15.2%) and EG-P (14.5%). This finding is noteworthy because the styrene sulfonate may capture a significant amount of water molecules during OECT operation, resulting in the swelling of the PEDOT:PSS film with water^50^, and thereby the possible deterioration of device stability (vide infra). Therefore, the above-mentioned results suggest that a substantial portion (i.e., 1.2 parts) of styrene sulfonate units were removed during the solvent-assisted crystallization, while the aggregates of PEDOT:PSS were rearranged to form a crystallized film with highly organized anisotropic ordering. Besides, the atomic force microscopy (AFM) and high-angle annular dark-field scanning transmission electron microscopy (HAADF-STEM) images shown in Supplementary Figure 3 reveal that the Crys-P film contains more uniformly distributed nanopores than the EG-P film. These results also suggest that the space occupied by PSS in the pristine PEDOT:PSS film becomes uniformly distributed with nanopores due to the removal of excessive PSS and the compact crystallization of the remaining PEDOT:PSS. Moreover, these uniformly distributed nanopores and residual PSS can act as water channels, which could permit the facile ion transport into highly crystallized PEDOT:PSS domains for dedoping PEDOT chains (vide infra).

### Electrical characterization of PEDOT:PSS-based OECTs

**Fig. 3:**
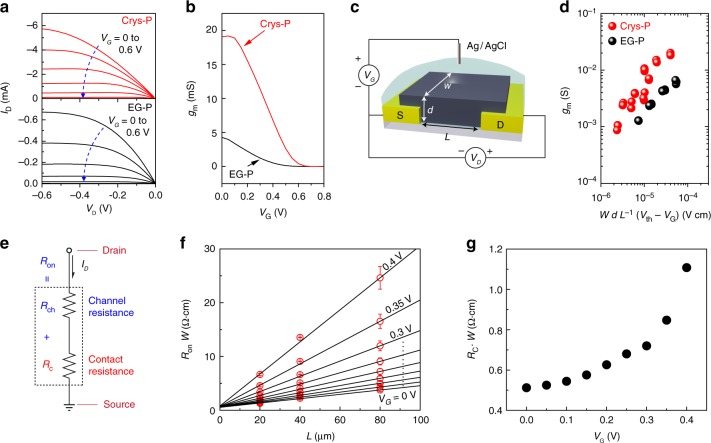
Electrical characterizations of Crys-P and EG-P OECTs. **a** Output and **b** transconductance (*g*_m_) characteristics of crystallized PEDOT:PSS (Crys-P, red curves) and 5% ethylene glycol-treated PEDOT:PSS (EG-P, black curves) OECT devices. In the output plots, *V*_G_ was scanned from 0 to 0.6 V along the blue dotted arrow. **c** a schematic definition of the geometrical parameters in the OECT channel. *W*, *d*, and *L* denote the width, thickness and length of the channels, respectively. **d** The scattered plots of Cyrs-P (red circles)/EG-P(black circles) *g*_m_ as a function of applied gate bias and channel geometry [*WdL*^−1^ (*V*_th_ – *V*_g_)]. Each data point represents an OECT measurement with a given channel geometry. **e** A equivalent circuit model for the discrimination between *R*_c_ and *R*_ch_ within a transistor channel. **f** TLM analysis of the Crys-P OECT (*d* = 61 nm) with the equivalent circuit model. **g** The plot of *W*-normalized *R*_c_ as a function of *V*_G_

In order to investigate the correlation between film microstructural crystallinity/composition and the consequent charge transport in ion-mediated electronic devices, OECTs were fabricated with Crys-P and EG-P films. Note that the electrical conductivities of Pr-P, EG-P, and Crys-P films are 1.7, 590, and 4100 S cm^−1^, respectively, which are comparable to the metrics reported in the previous literature^9,19,38^. (Supplementary Table 1) Briefly, PEDOT:PSS films were deposited on top of quartz substrates with pre-patterned gold source/drain electrodes, and an active channel with a width (*W*) of 80 μm and a length (*L*) of 20 μm as well as SU-8 passivation were defined via conventional photolithography (Supplementary Fig. 4a). Note that the H_2_SO_4_ treatment for Cry-P formation did not affect the integrity of metallic electrode patterns, which could be ascribed to very slow diffusion of viscous H_2_SO_4_ into Cr adhesive layer (5 nm) below inert Au film (50 nm) as shown in Supplementary Figure 4b. An aqueous electrolyte solution was placed in contact with the channel region using a polydimethylsiloxane well, while a suspended Ag/AgCl wire served as a non-polarizable gate electrode^51,52^. Figure 3a shows the output characteristics of OECTs based on Crys-P (200 nm) and EG-P films (190 nm). Interestingly, the maximum drain current (*I*_D_) in Crys-P is ~ 10 times larger than that of EG-P, although the PEDOT:PSS layer with similar film thickness was deposited for the OECT channel. Both devices exhibit typical pinch-off behavior while the drain current (*I*_D_) decreases with an increased gate voltage (*V*_G_) from 0 to 0.6 V, which is attributed to dedoping in the PEDOT:PSS channel^8^. As shown in Supplementary Figure 5, saturation-regime transfer curves plotted with the drain voltage (*V*_D_) fixed at −0.6 V also confirm an enhanced drain current in Crys-P at the on-state gate bias range (0 < *V*_G_ < 0.6); however, the drain current difference between Crys-P and EG-P devices becomes marginal at the off-state (*V*_G_ > 0.6 V). It is also noteworthy that, in contrast to the channel current, the gate current (*I*_G_) in Crys-P OECT was comparable to that of EG-P device. To examine the efficiency of channel current modulation at a given gate bias, the differential transconductance (*g*_m_) was extracted from each transfer curve shown in Supplementary Figure 5, since this value is one of the most widely accepted figures-of-merits in OECT and other transistor-based sensors (Fig. 3b)^39^. Crys-P shows a maximum transconductance near *V*_G_ = 0 V of 19 mS, which well exceeds that of EG-P (4 mS), as well as those estimated from other OECT devices with similar channel dimensions reported in the literature^39^. Note also that the observed maximum transconductance at *V*_G_ = 0 V could be beneficial for designing a simplified amplifying transducer for a variety of biomedical devices^53^. The excellent performance of Crys-P OECT in terms of large on-state current, low off-state/leakage current, and high transconductance can be justified by the above mentioned channel material’s microstructure and composition, i.e., highly ordered π–π and edge-on stacking in combination with a relatively large PEDOT content (due to relatively low PSS inclusion), which facilitates charge carrier transport in the OECT channel.

The volumetric nature of the charge-transporting channel is understood to be responsible for its unique OECT operation mechanism, distinguished from other types of transistors operating in the field-effect mode;^54,55^ therefore, there arises a need to consider the effective channel thickness (*d*) as an explicit parameter for the charge transport model^29^. Based on benchmarking by Inal et al. of various organic mixed conductors, the product of charge carrier mobility and volumetric charge storage capacity ([µC^*^]) can be employed as a material/system figure-of-merit^56^. Therefore, various Crys-P and EG-P OECTs were quantitatively analyzed by collecting their saturation-regime transfer curves while their channel dimensions (i.e., width, length and thickness) were varied (Fig. 3c). The linearity between *g*_m_ and *W d L*^−1^ (*V*_th_ − *V*_G_) in our OECTs is well visualized in Fig. 3d, leading us to extract the proportionality factor [μC^*^] as 490 ± 41 and 100 ± 7 F cm^−1^V^−1^s^−1^ for Crys-P and EG-P OECTs, respectively. Considering that the highest value reported among PEDOT-based materials or all mixed organic conductors is 72 from PEDOT:Tos^56^ or 261 F cm^−1^V^−1^s^−1^ from p(g2T-TT)^23^, this much higher value from our Crys-P suggests that its unique anisotropic edge-on alignment, relatively high PEDOT inclusion, and enhanced crystallinity/nanoporosity may effectively contribute to the balanced mixed conduction and, thereby, the unprecedentedly high [μC^*^] value (see Supplementary Fig. 6 and Supplementary Table 2).

### Contact resistance in PEDOT:PSS OECTs

Since Crys-P OECTs exhibit very high on-state current (at *V*_G_ = 0 V) due to the relatively high conductivity of Crys-P^38^, in principle, the contact resistance at a metal/Crys-P junction can negatively affect the resultant OECT performance. Furthermore, it could be difficult to achieve ideal ohmic contact at a metal/organic junction due to widely occurring Fermi-level pinning and deviation from the Mott-Schottky model^57,58^. In this regard, insufficient charge injection as well as contact resistance may seriously limit channel current in organic devices, especially in short-channel transistors^59,60^. Therefore, we analyzed the contact resistance in an OECT with the bottom gold source/drain contact on Crys-P, by applying the transmission-line method (TLM), assuming that the total source-to-drain channel resistance (*R*_on_) is equivalent to the sum of channel resistance (*R*_ch_) and contact resistance (*R*_c_) in series^61^ (Fig. 3e). First, as shown in Fig. 3f, the total channel resistance at the given gate bias was extracted from each transfer curve measured in the linear regime, and the *W*-normalized *R*_c_ (*R*_c_·*W*) was determined with the zero-*L* limit value from the linear-extrapolated *L*-dependent *R*_on_·*W* plot. Then, the *V*_G_-dependent *R*_c_·*W* was drawn in Fig. 3g. The low contact resistance at *V*_G_ = 0 V can be understood as the result of mitigating contact depletion in the presence of a large density of free charge carriers at the minimal dedoping condition^62^. Although OECTs typically exhibit relatively lower contact resistance than solid-state OFETs by orders of magnitude^60^, Crys-P OECT exhibits exceptionally low values of *R*_c_·*W* (~ 0.5 Ω·cm) at *V*_G_ = 0 V, which is even smaller than those in ion-gel-gated polymer transistors^63^ and other aqueous-electrolyte OECTs (~ 2.5 Ω·cm)^64^. Such a low *R*_*c*_ value demonstrates another big advantage of Crys-P for OECTs and related bioelectronics, which require substantially scaled-down channel dimensions, particularly for a single cell-level or small-quantity biomolecular detection.

### Volumetric capacitance of PEDOT:PSS films

**Fig. 4:**
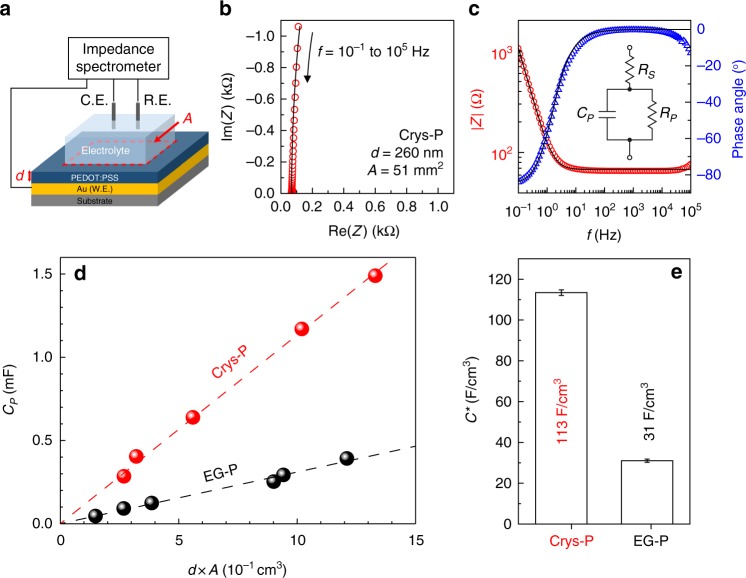
Electrochemical properties of Crys-P and EG-P films. **a** A schematic diagram of electrochemical impedance spectroscopy (EIS) measurements (C.E.: counter electrode, R.E. reference electrode (Ag/AgCl), and W.E: working electrode (PEDOT:PSS on Au). The film area and thickness are denoted by ‘*A’* and ‘*d’*, respectively. **b** Nyquist and **c** Bode plots acquired using the crystallized PEDOT:PSS (Crys-P) film as a working electrode. The EIS data (symbols) was fitted (black line) with an equivalent circuit model composed of a serial resistor (*R*_S_), a parallel resistor (*R*_P_), and a parallel capacitor (*C*_P_). **d** Plots of extracted parallel capacitance values as a function of active volume of Crys-P (red circles) and EG-P 5% ethylene glycol-treated PEDOT:PSS (black circles) films. **e** A bar plot of the volumetric capacitance values which were calculated from the slopes of *C*_P_ vs. *d×*A plots in Fig. 4d

Next, volumetric capacitance was extracted by using three electrode-based electrochemical impedance spectroscopy (EIS) to examine ion-mediated carrier modulation in Crys-P and EG-P films (Fig. 4a). Raw EIS data sets were analyzed with an equivalent circuit model composed of a serial resistor, parallel resistor, and parallel capacitor (Fig. 4b, c). Subsequently, each extracted parallel capacitance (*C*_*P*_) value was plotted as a function of the nominal volume of the PEDOT:PSS film or the product of film thickness (*d*) and area (*A*). First, in both Crys-P and EG-P films, the extracted capacitances were linearly proportional to the film volumes (*d* · *A*). As is well documented by Malliaras and coworkers, there exists a linear relationship between electrochemical capacitance and film volume, and the volumetric capacitance can be estimated from the (constant) slope of the film capacitance vs. volume plot^29,65^. The key findings are described as follows. First, as shown in Fig. 4d, the volumetric response of electrochemical capacitance is confirmed in both EG-P (black circles) and Crys-P devices (red circles), suggesting that the highly crystalline microstructure and the PSS-deficient composition in Crys-P do not prevent aqueous ions from permeating into the solid PEDOT:PSS matrix throughout the whole film thickness. Secondly, the Crys-P film shows much higher volumetric capacitance (113 F cm^−3^) than the EG-P film (31 F cm^−3^, Fig. 4e). This result implies that a substantially large volume density of charges could be accommodated throughout the Crys-P film, which can be ascribed to the relatively high content of PEDOT units in Crys-P after a substantial number of excessive PSS chains are removed. Third, the actual value of volumetric capacitance (C^*^) extracted from Crys-P film is comparable to that of the highly ordered PEDOT:Tos film (~136 F cm^−3^)^66^, implying that it is possible to prepare high-performance OECTs using the simple solution-processed Crys-P film.

### Aqueous and thermal stability of PEDOT:PSS OECT devices

**Fig. 5:**
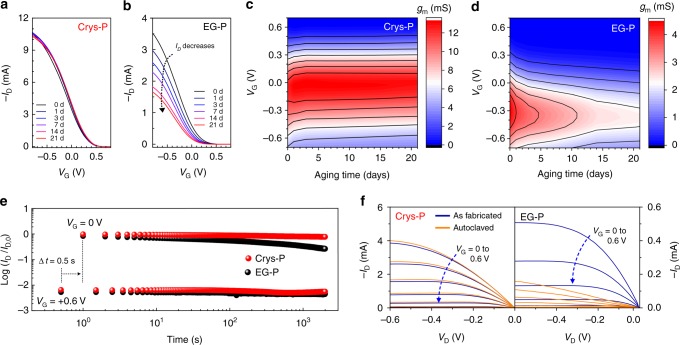
Aqueous stability of Crys-P and EG-P OECTs. **a** The saturation-regime transfer curves of crystallized PEDOT:PSS (Crys-P) and **b** 5% ethylene glycol-treated PEDOT:PSS (EG-P) OECTs over aging time in an aqueous 0.1 M NaCl solution. **c** The color-coded contour plots of transconductance (*g*_m_) of Crys-P and **d** EG-P films measured at the same condition as in (**a**, **b**). **e** Normalized current on/off ratio traced during the repeated *V*_G_ switching up to 2000 cycles (*V*_G_ *=* 0.6 V [off] and 0 V [on], *∆t* = 0.5 s). **f** Output characteristics of Crys-P (left panel) and EG-P (right panel) OECTs before (blue curves) and after (orange curves) autoclave sterilization while *V*_G_ was swept from 0 to 0.6 V with the increment of 0.1 V

There exist several advantages of OECT-based devices; in particular, their low-voltage operation with high on-off current ratio in the presence of aqueous electrolytes has inspired many researchers to employ OECTs as a platform technology for disposable biomolecule sensors and implantable brain signal recorders^13,17,18,25^. For practical application of such devices, the long-term aqueous stability of the material itself and device operation should be seriously considered, but little attention has been paid to these issues up to now. Therefore, we performed device stability tests under three representative stress conditions: (i) long-term aging, (ii) repeated gate bias switching, and (iii) thermal shock in autoclave sterilization. For these tests, OECTs with a channel width of 80 μm and length of 20 μm were prepared using Crys-P and EG-P films of the very similar thickness (~ 100 nm). First, the aging test was conducted by immersing both devices in 0.1 M NaCl at 37 °C for a designated period, followed by collecting transfer characteristics (*V*_*D*_ = −0.6 V). As shown in Fig. 5a, b, the on-state *I*_D_ of Crys-P OECT was not substantially changed even after 21-day immersion, whereas that of EG-P OECT was gradually reduced down to half of the initial on-current after the same-period of immersion. In the case of the off-state *I*_D_, both devices exhibited very stable behavior during the aging test. These trends are even clearer in Fig. 5c, d, where each transconductance curve is presented in a color-coded contour map as a function of aging time. Remarkably, Crys-P OECTs show a maximum transconductance of ~ 13 mS near 0 V and this feature remains almost the same over time even after 21-day immersion. This is in contrast to EG-P devices, where the maximum transconductance near −0.3 V gradually decreases from 4.5 (0 day) to 2.3 mS (21 day). We suspect that the apparent degradation of OECT performance could originate from swelling of the EG-P film; thus, the reduction in channel conductivity is due to the excessive PSS residues in the film (see Fig. 2)^67^. For clarification, the thicknesses measured from dry Crys-P and EG-P films were compared with those measured from the corresponding wet films using liquid-cell AFM under water (Supplementary Fig. 7). Interestingly, the Crys-P film maintained its original thickness within 20% increase after water dipping, but the EG-P film showed a 480% increase of thickness. It is also noteworthy that the chemically cross-linked EG-P film also showed the substantial increase in film thickness (220%) after water immersion, although chemical cross-linking enables the stable operation of PEDOT:PSS devices in water. Such a small swelling ratio in the Crys-P film can be attributed to a three-times-lower content of styrene sulfonate unit as well as a higher polymer crystallinity than in the EG-P film. This result also indicates that the unique film microstructure and composition in Crys-P results in long-term aqueous stability of the PEDOT:PSS film itself and the corresponding OECT operation.

As a stress test under the condition of repeated gate bias switching, the on- and off-state channel currents (*I*_D_) were monitored while voltages of + 0.6 and 0 V were periodically applied on the gate electrode (*V*_D_ = −0.6 V, *∆t* = 0.5 s). As shown in a plot of log (*I*_D_ / *I*_D,0_) vs. time (Fig. 5e), Crys-P OECTs exhibit stable on- and off-state values even after 2000 cycles, whereas EG-P OECTs exhibit a significant decrease in on-state *I*_D_ over time. More specifically, the initial on-state *I*_D_ value of EG-P OECT (*V*_D_ = −0.6 V, *V*_G_ = 0 V) is reduced by ~ 75% after 2000 cycles (equivalent to 40 min), which is substantially larger than the on-state *I*_D_ reduction (*V*_D_ = −0.6 V, *V*_G_ = 0 V) by ~ 25% after immersion of EG-P OECT in water for 1 day (Fig. 5b). This indicates that EG-P devices are more susceptible to repeated bias stress under prolonged water immersion. Finally, a thermal shock test was conducted on Crys-P and EG-P OECTs. Autoclaving using high-pressure saturated steam treatment at high temperature (2 atm, 125 °C for 15 min) is one of the most conventional and reliable methods for sterilizing biomedical devices^68^, and stable/reliable device performance after such a harsh treatment is highly desired for extending OECT applications to human-body implantable bioelectronics. Figure 5f reveals the output characteristics of Crys-P and EG-P OECTs before (blue curves) and after (orange curves) autoclaving. Although EG-P OECT shows a significant decrease in overall *I*_D_ values after autoclaving, the Crys-P device does not display such a phenomenon. Careful examination determined that autoclaving slightly increased overall *I*_D_ values, which might imply that the film microstructure in Crys-P underwent an additional improvement under the high temperature and pressure condition. Furthermore, no significant change in the Crys-P OECT transfer curves demonstrates that all devices can operate stably over the entire range of gate biases regardless of the heat/pressure shock (Supplementary Fig. 8). Considering that PEDOT:PSS devices fabricated without chemical cross-linking do not survive after autoclave sterilization, it is remarkable that the crystallized PEDOT:PSS (Crys-P) showed the stable OECT performance after autoclave sterilization^68^. In other words, the essential features of PEDOT:PSS films, such as electrical conductivity, reversible ion doping/dedoping, and thereby its excellent OECT performance, are not seriously affected by autoclaving once the microstructure and composition of the active channel material is properly manipulated. It is typically accepted that most organic electronic material-based devices are highly susceptible to common environmental factors such as temperature, pressure, and humidity. Indeed, there exist very few reports of small molecule- or polymer-based electrical devices which exhibit significant robustness to autoclaving^69^. We propose that solvent-assisted crystallization of PEDOT:PSS, which results in structural rearrangement (i.e., highly crystalline edge-on stacking) and modified composition (i.e., removal of excessive styrene sulfonate units) (see Fig. 2), substantially contribute to the material and device robustness against repeated bias stress, prolonged water immersion, and even thermal/pressure shock.

## Discussion

We investigated the correlation among film microstructural crystallinity/composition, electrochemical transistor performance, and aqueous stability in various poly(3,4-ethylenedioxythiophene):poly(styrenesulfonate) (PEDOT:PSS) films. We demonstrated that the post-treatment of an as-spun PEDOT:PSS film with concentrated sulfuric acid leads to an unconventional PEDOT:PSS film microstructure as represented by anisotropic polymer microstructure (i.e., vertical edge-on and horizontal π–π orderings) with enhanced crystallinity and nanoporosity. More importantly, even though the Crys-P film exhibits a highly ordered film microstructure, cations can still access the PEDOT chains throughout the whole film for dedoping due to nanoscale pore formation and uniform PSS dispersion, leading to 3.6-times larger volumetric capacitance (113 F cm^−2^) than EG-P. To the best of our knowledge, this is the highest value reported among PEDOT:PSS-based materials. Owing to the above mentioned film microstructure and composition of Crys-P, the corresponding OECTs show unprecedented electrical performance and aqueous stability. For instance, Crys-P OECTs showed ~ 10 times higher on-state *I*_D_ and 4 times larger maximum transconductance than EG-P OECTs. Furthermore, Crys-P has the highest OECT benchmark value (*i.e*., [μC^*^]) of 490 ± 41 F cm^−1^V^−1^s^−1^ and a low contact resistance below 1 Ω cm^−1^. In addition, an extraordinary tolerance of Crys-P OECTs to harsh conditions such as long-term water immersion, repeated gate bias switching, and autoclaving-based sterilization was demonstrated for the first time. Note that even the introduction of chemical cross-linkers into the PEDOT:PSS layer by adding 3-glycidyloxypropyl)trimethoxysilane (GOPS) cannot sufficiently prevent the deterioration of electrical conductivity by water immersion^31^. Based on these results, we suggest that Crys-P provides highly ordered crystallized polymer microstructure in combination with nanoscale pores, which are beneficial not only for efficient hole transport in the horizontal channel, but also for unblocked ion permeation into conjugated moieties in the vertical direction. Simultaneously, minimal hydrophilic PSS residues attenuate the volume change of the film, thereby affording a robust operational stability in prolonged contact with aqueous electrolytes. Taken together, our study clearly demonstrates that there exists a strong correlation among film microstructural crystallinity/composition, electrochemical transistor performance, and aqueous stability in PEDOT:PSS films. Furthermore, we expect that our results will contribute to further understanding of the fundamental aspects of ion/hole-mixed transport at the channel-electrolyte interface, and to the use of highly crystallized PEDOT:PSS devices for implantable bioelectronics targeted at chronic neural recording and stimulation.

## Methods

### Film preparation and characterizations

Crys-P films were prepared as reported in the previous literature^38^. Pristine PEDOT:PSS films were deposited by spin-coating on pre-cleaned substrates and annealed at 120 °C for 15 min after the aqueous PEDOT:PSS solution (Clevios PH1000, Heraeus) was filtered using cellulose acetate syringe filters (0.45 μm pore size, Advantec MFS, Inc.) prior to use. For Crys-P films, pristine films were left in a bath of concentrated sulfuric acid (> 95%, Duksan Pure Chemicals) for 15 min, thoroughly rinsed with deionized water, and dried at 120 °C for 15 min. To prepare EG-P films, the PEDOT:PSS solution (2 mL) mixed with ethylene glycol (0.5 mL, Sigma Aldrich) and dodecylbenzene sulfonic acid (5 μL, Sigma Aldrich) was spin-cast onto PET substrates, and dried at 120 °C for 15 min. To prepare chemically cross-linked EG-P films, 1 wt% of 3-glycidyloxypropyl)trimethoxysilane was additionally added to the EG-P precursor solution, and corresponding films were fabricated in the above mentioned manner. For X-ray measurement, all PEDOT:PSS films were prepared on p-Si^++^/SiO_2_ (300 nm) substrates. Wide angle GIWAXS was performed at Stanford Synchrotron Radiation Lightsource (SSRL, beamline 11–3) with the photon energy of 12.7 keV. The incidence angle α of the incident beam was set to 0.1°, above the critical thickness of the polymer film but below that of the silicon substrate. The diffraction intensity was detected with a 2D CCD detector (Rayonix MAR-225). The XPS spectra were obtained using a K-alpha spectrometer (Thermo Scienctific Inc.) with Al-K_α_ radiation. The HAADF-STEM images were acquired using a Tecnai G^2^ F30 S-Twin microscope operated at 300 kV. The TGA curves were obtained in the range 30–700 °C at 3 °C/min using a PerkinElmer TGA 4000. The electrical conductivities of various PEDOT:PSS thin films were measured by a four-point probe method using Keithley 2400.

### EIS measurements

All PEDOT:PSS films were prepared on Au-coated silicon substrates as working electrodes, while the electrode surface other than the active area was passivated using epoxy glue. Electrochemical impedance spectra were obtained in the 0.1 M NaCl solution using PGSTAT304N (Metrohm Autolab, the Netherlands) equipped with a conventional three-electrode system composed of a working electrode, an Ag/AgCl reference electrode, and a Pt counter electrode, at the frequency range between 0.1 and 10^5^ Hz with a single sinusoidal signal of *E*_ac_ = 25 mV at *E*_dc_ = 0 V.

### AFM measurements

Regarding the swelling test, film thickness was measured using a Park Systems XE-Bio AFM equipped with a 10 nm radius tip cantilever (PPP-CONTSCR, Nanosensors). The circular pattern with the radius of 10 μm was defined on PEDOT:PSS films by the conventional photolithography and scanned by AFM in the contact mode. The topographic images were obtained with a spatial resolution of 512 × 512 points and a scanning area of 90 × 90 μm. To evaluate the thickness change in Crys-P and EG-P films after water immersion, the same circular patterns were scanned in the dry condition and re-scanned in the liquid cell after the 20-min immersion in water.

### OECT fabrication and characterizations

All OECT devices were fabricated via the conventional photolithographic technique. Source and drain electrode patterns (Cr (5 nm)/Au (50 nm)) were prepared on a pre-cleaned p-Si^++^/SiO_2_ (300 nm) substrates using a positive photoresist (GXR-601, Microchemicals GmbH). Crys-P or EG-P channels were fabricated by depositing each film on the electrode-patterned substrate, followed by preparing a positive photoresist pattern and dry-etching the POEDOT:PSS films at the non-channel area. After the residual photoresist removal, the outer electrode patterns were passivated using SU-8 photoresist (Microchemicals GmbH). A polydimethylsiloxane (PDMS, SYLGARD^®^ 184, Dow Corning) ring was attached to define the aqueous electrolyte reservoir. All electrical measurements were conducted using a Keithley 4200A-SCS parameter analyzer (Keithley, USA) while an Ag/AgCl reference electrode was employed as a gate electrode. For aging test, OECTs immersed in the solution of 0.1 M NaCl were placed in a biological CO_2_ incubator (ThermoFisher Scientific) with 5% CO_2_ at 37 °C. The autoclaving test was evaluated after OECT devices were placed in a Tomy SS-325 autoclave during a single sterilization cycle.

## Electronic supplementary material

Supplementary Information

## Data Availability

The data that support the findings of this study are available from the corresponding author upon reasonable request.
