# Supplementary material for: Influence of PEDOT:PSS crystallinity and composition on electrochemical transistor performance and long-term stability
Source: Nat Commun. 2018 Sep 21;9:3858. doi: 10.1038/s41467-018-06084-6 (PMC6155079; doi:10.1038/s41467-018-06084-6)
Supplement: Supplementary file 1 — Supplementary Information [file 41467_2018_6084_MOESM1_ESM.pdf]

Supplementary Information

Correlation Among PEDOT:PSS Film Microstructural Crystallinity/Composition, Electrochemical Transistor Performance, and Long-Term Stability

Seong-Min Kim[1,†], Chang-Hyun Kim[1,2,†], Youngseok Kim[1], Nara Kim[1], Won-June Lee[1], Eun-Hak Lee[1], Dokyun Kim[1], Sungjun Park[3], Kwanghee Lee[1,2,4], Jonathan Rivnay[5], and Myung-Han Yoon[1,*]

[1]School of Materials Science and Engineering, Gwangju Institute of Science and Technology, Gwangju 61005, Republic of Korea

[2]Research Institute for Solar and Sustainable Energies, Gwangju Institute of Science and Technology, Gwangju 61005, Republic of Korea

[3]RIKEN Center for Emergent Matter Science (CEMS), 2-1 Hirosawa, Wako, Saitama, 351-0198, Japan

[4]Heeger Center for Advanced Materials, Gwangju Institute of Science and Technology, Gwangju 61005, Republic of Korea

[5]Department of Biomedical Engineering, Northwestern University, Evanston, IL 60208, USA

[†]These authors contributed equally to this work.

[*]E-mail: mhyoon@gist.ac.kr

This Supplementary Information includes: Supplementary Figures 1 to 8 and Supplementary Tables 1 to 2

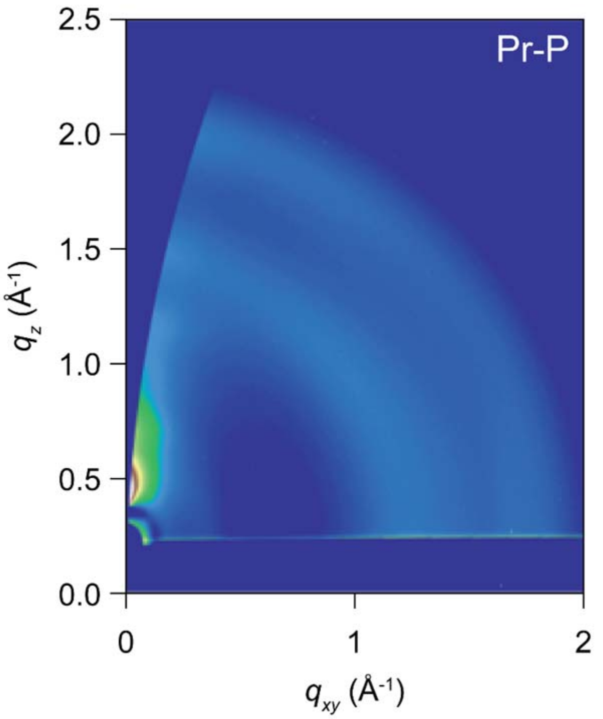

Supplementary Figure 1. Grazing incidence wide-angle x-ray scattering (GIWAXS) patterns of a pristine PEDOT:PSS (Pr-P) film.

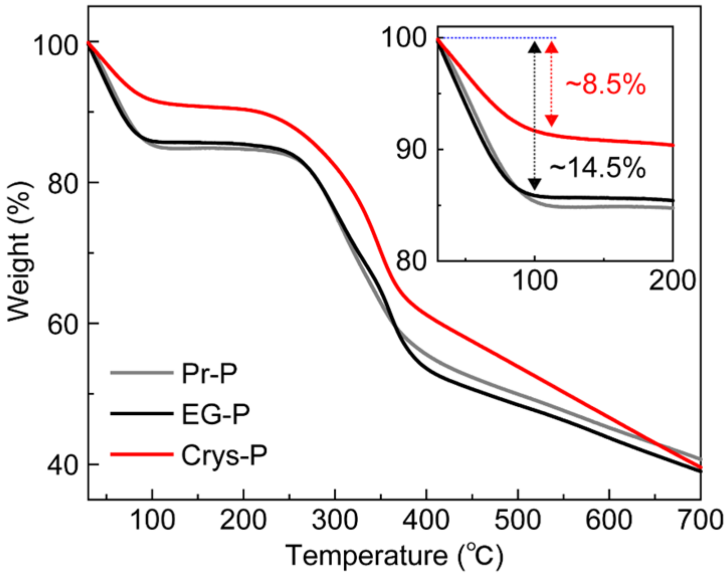

Supplementary Figure 2. Thermogravimetric analysis (TGA) curves of PEDOT:PSS films. The inset shows magnified plots at the low-temperature region.

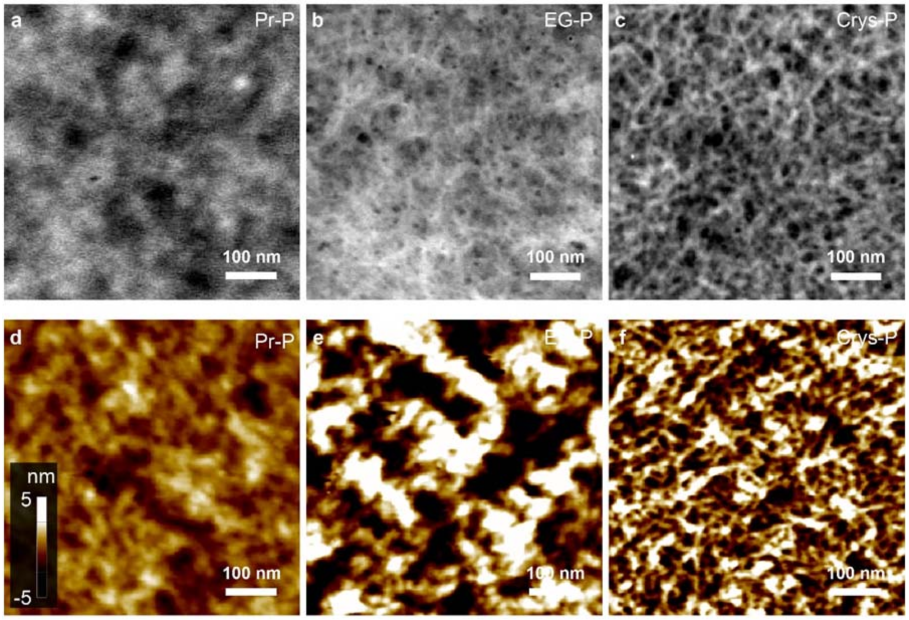

Supplementary Figure 3. The microscopic morphologies of PEDOT:PSS films (a - c) High-angle annular dark-field scanning transmission electron microscope (HAADF-STEM) and (d - f) Atomic force microscope (AFM) images showing the surface nanostructures of Pr-P, EG-P, and Crys-P films.

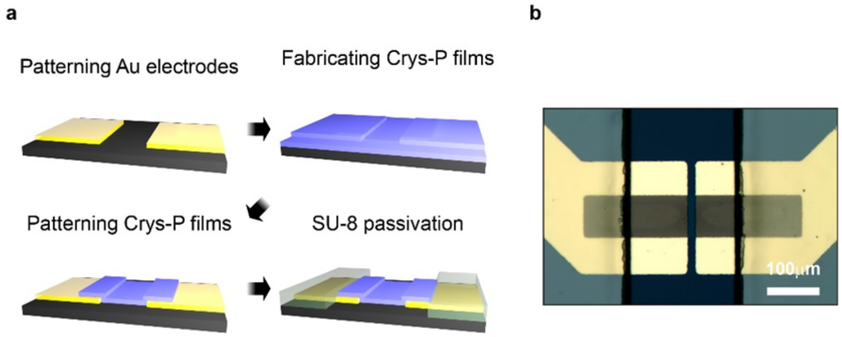

Supplementary Figure 4. Fabrication of PEDOT:PSS organic electrochemical transistor (OECTs) (a) Fabrication procedures for crystallized PEDOT:PSS (Crys-P) OECTs and (b) the optical micrograph of Crys-P OECTs after PEDOT:PSS layer patterning and SU-8 passivation.

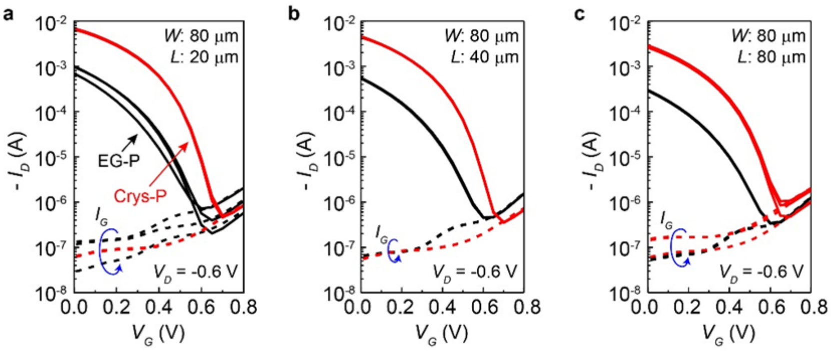

Supplementary Figure 5. Saturation-regime transfer characteristics of EG-P (black) and Crys-P (red) OECT devices with various channel length while V_D was fixed at -0.6 V.

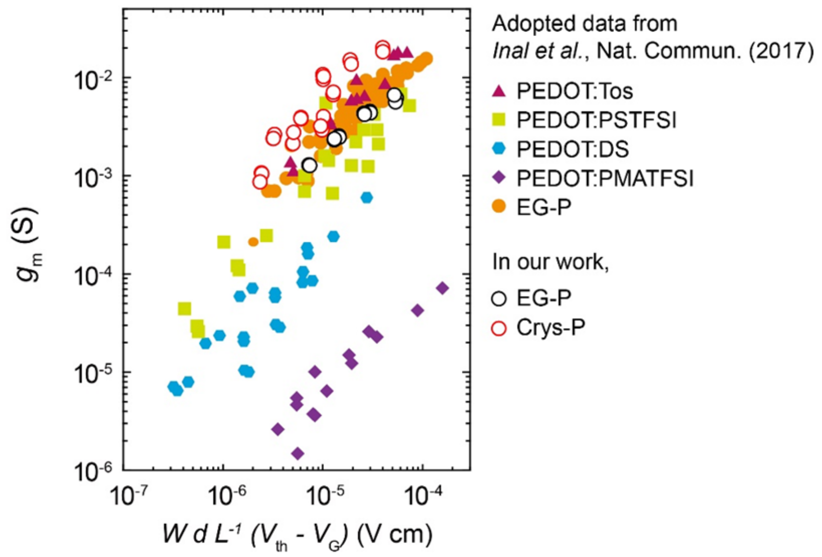

Supplementary Figure 6. Transconductance (g_m) of OECTs as a function of operation voltage and channel geometry. The OECT data from PEDOT:Tos, PEDOT:PSTFSI, PEDOT:DS, PEDOT:PMATFSI, and EG-P were adopted from [1].

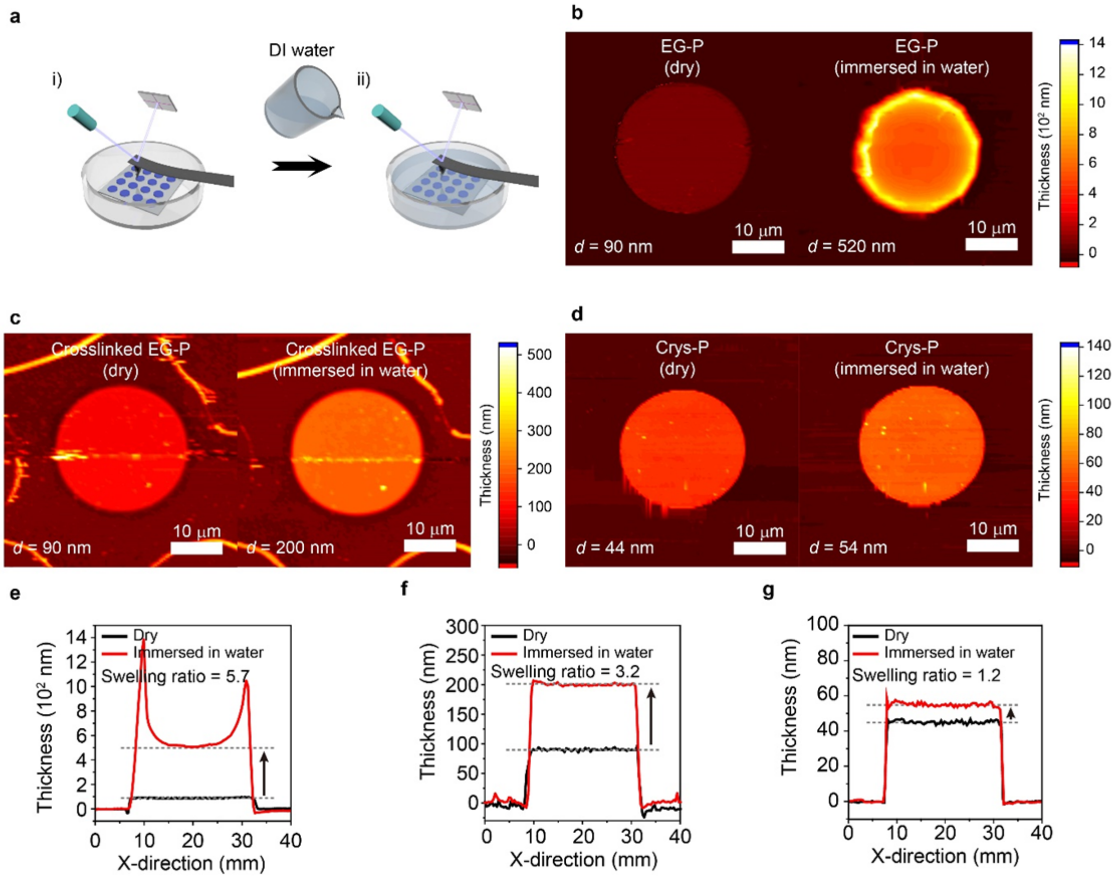

Supplementary Figure 7. The film swelling of PEDOT:PSS films (a) A schematic of measuring the film swelling ratio based on dry and liquid AFM measurements. Comparison of the difference between dry and wet film thickness in (b, e) EG-P, (c, f) chemically crosslinked EG-P, and (d, g) Crys-P. The circular patterns of PEDOT:PSS films were generated by photolithography and scanned by either dry and liquid AFM. The swelling ratio is calculated by diving wet film thickness by dry film thickness.

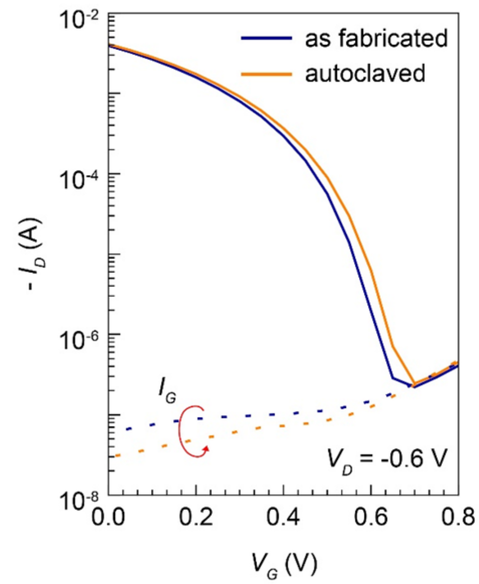

Supplementary Figure 8. Transfer characteristics of Crys-P OECTs before and after autoclaving.

Supplementary Table 1. Electrical conductivities of various PEDOT:PSS films

Materials	Electrical conductivity (S/cm)*
Pr-P	1.7 ± 0.12
EG-P	590 ± 20
Crys-P	4100 ± 98

* The average of three sample metrics in conjunction with the corresponding standard deviation were presented.

Supplementary Table 2. The figure-of-merits of OECTs made of PEDOT-based materials.

Materials/Formulation	C^* (F cm^{-3})	$[\mu C^*]_{OECT}$ (F cm^{-1}V^{-1}s^{-1})
PEDOT:Tos	136 ± 50	72 ± 14
PEDOT:PSTFSILi100	26 ± 10	20 ± 1.6
PEDOT:DS + EG	65 ± 46	2.2 ± 0.9
PEDOT:PMATFSILi80	27 ± 7	0.15 ± 0.01
PEDOT:PSS + EG	39 ± 3	47 ± 6
EG-P (Our result)	31 ± 0.79	100 ± 7
Crys-P	113 ± 1.38	490 ± 41

The volumetric capacitance and $[\mu C^*]$ data of PEDOT:Tos, PEDOT:PSTFSILi100, PEDOT:DS + EG, PEDOT:PMATFSILi80, and PEDOT:PSS + EG are adopted from [1]. Tosylate (Tos), PSTFSILi100, dextran sulfate (DS), PMATFSILi80, and poly(styrene sulfonate) (PSS) are various molecular, polymeric, and biological anionic dopants complexed with PEDOT. Ethylene glycol (EG) is a co-solvent additive known to improve electronic conductivity of polymer films.

Supplementary References

1. Inal, S., Malliaras, G. G. & Rivnay, J. Benchmarking organic mixed conductors for transistors. *Nat. Commun.* **8,** 1767 (2017).
